# Hepatitis E Virus in Manure and Its Removal by *Psychrophilic anaerobic* Biodigestion in Intensive Production Farms, Santa Catarina, Brazil, 2018–2019

**DOI:** 10.3390/microorganisms8122045

**Published:** 2020-12-21

**Authors:** Doris Sobral Marques Souza, Deisi Cristine Tápparo, Paula Rogovski, Rafael Dorighello Cadamuro, Estêvão Brasiliense de Souza, Raphael da Silva, Roberto Degenhardt, Juliano De Dea Lindner, Aline Viancelli, William Michelon, Airton Kunz, Helen Treichel, Marta Hernández, David Rodríguez-Lázaro, Gislaine Fongaro

**Affiliations:** 1 Laboratory of Applied Virology, Department of Microbiology, Immunology and Parasitology, Federal University of Santa Catarina, Florianópolis, SC 88040-900, Brazil; doris.sobral@gmail.com (D.S.M.S.); paularogovski@gmail.com (P.R.); rafaelcada@hotmail.com (R.D.C.); ebsouza@gmail.com (E.B.d.S.); rafasilva1993@gmail.com (R.d.S.); 2 Department of Food Science and Technology, Federal University of Santa Catarina, Florianópolis, SC 88034-001, Brazil; robertodegenha@gmail.com (R.D.); julianoddlindner@ufsc.br (J.D.D.L.); 3 UNIOESTE/CCET/PGEAGRI, Western Paraná State University, Cascavel, PR 85819-110, Brazil; deisictapparo@gmail.com; 4 PMPECSA-UnC, University of Contestado, Concórdia, SC 89711-330, Brazil; alinevbortoli@gmail.com (A.V.); william@unc.br (W.M.); 5 Embrapa Suínos e Aves, Concórdia, SC 89715-899, Brazil; airtonkunz@gmail.com; 6 Laboratory of Microbiology and Bioprocesses, Federal University of Fronteira Sul, Erechim, RS 99700-970, Brazil; helen.treichel@uffs.br; 7 Instituto Tecnológico Agrario de Castilla y León, 47071 Valladolid, Spain; hernandez.marta@gmail.com; 8 Division of Microbiology, Department of Biotechnology and Food Science, Universidad de Burgos, 09001 Burgos, Spain

**Keywords:** Hepatitis E virus, zoonotic pathogens, enteric viruses, pig production circle, psychrophilic anaerobic biodigesters, virus inactivation, food safety

## Abstract

Hepatitis E virus (HEV) is an important enteric agent that can circulate in swine; it is excreted in manure, and of zoonotic interest. The present study investigated, by RT-qPCR, the circulation of HEV in swine manure from different types of pig farms (maternity, nursery, and grow-finish farms) in Santa Catarina State, the major pig production area of Brazil, and also evaluated the HEV removal efficiency of psychrophilic anaerobic biodigesters (PABs). While HEV was consistently detected in manure from grow-finish pig farms (>4 log HEV genome copies (GC) L^−1^), the virus was not detected in manure from maternity and nursery farms. These findings suggest a potential high biosafety status during primary-swine production, with a subsequent contamination in grow-finish production. The anaerobic biodigestion process reduced more than 2 log_10_ HEV GC in the processed swine manure. However, the virus concentration in final effluent remained high, with an average value of 3.85 log_10_ HEV GC L^−1^. Consequently, our results demonstrate that PABs can be a robust tool for effective inactivation of HEV, while reinforcing the need for sanitary surveillance and legislation of swine manure-derived biofertilizers, to avoid the spread of zoonotic enteric pathogens such as HEV.

## 1. Introduction

Swine farming produces high amounts of waste (faeces, urine) that can contain numerous animal and human pathogens, which can reach the environment and water sources. A method widely used for treatment and reuse of swine manure is psychrophilic anaerobic biodigestion (PAB) [1,2,3]. PABs operate continuously, with hydraulic retention time between 30 and 50 days, with the efficiency of the digestion process dependent on natural temperatures, since these systems are not heated [3,4]. The digested material contains nitrogen, phosphorus, and potassium and is used as an organic fertilizer [5]. In large-scale production, efficient management of the waste generated is essential, as it can be a source for the spread of infectious agents, some of them zoonotic, such as hepatitis E virus (HEV) [6]. Indeed previous evidence has shown a robust detection of HEV in pig by-products plants (up to 80%), mainly in the first stages of the process [7]. Brazil is the fourth major exporter of pigs and derived products worldwide [8], and slaughtered more than 11 million pigs in the last quarter of 2019 [9]. Santa Catarina State is the major Brazilian pig producer and exporter [8]. HEV circulation has been already reported in Brazil in both humans, domestic pigs, and the environment [10]. The present study aimed to evaluate the presence of HEV in swine manure along pig production system (swine maternity, swine nursery, and grow-finish swine production farms), as well as to evaluate the efficiency of PABs in the removal of HEV.

## 2. Materials and Methods

### 2.1. Manure Sampling

Different cycles of swine system chain were selected in an area of intense production of swine manure in February 2018 and March 2019: three farms specialized in swine maternity (400 breeding females); three farms specialized in swine nursery (600 young animals); and three farms specialized in grow-finish swine (400 animals). All farms are located in Concórdia city, Santa Catarina State, Brazil (27°18′ S, 51°59′ W). All production systems had PABs for swine manure treatment, with recycling of the waste as fertilizer in pasture areas.

Five samples of 200 mL were taken in each sampling event, and were thoroughly mixed. Subsequently, 3 aliquot of 25 mL of each pool were taken for subsequent RT-qPCR analysis, and were processed independently.

### 2.2. Detection of HEV by Real-Time PCR

#### 2.2.1. Sample Process Control Virus

Simian Rotavirus—SA11 (group A, serotype G3), propagated in MA104 cells, was used as a sample process control virus (SPCV) [11,12], and added to each sample immediately before the start of the analysis.

#### 2.2.2. Virus Concentration and Nucleic Acid Extraction from Manure

RNA was extracted from 25 mL of manure samples as previously described by Viancelli et al. [12]. Briefly, 25 mL of sample was clarified and concentrated using glycine buffer method coupled with polyethylene glycol precipitation. Nucleic acid extraction was performed using a QIAmp MinElute Virus Spin Kit (Qiagen, Germany) following the manufacturer’s instructions. A negative process control (NPC) containing the reagents used for concentration and extraction of the sample, and the SPCV, but without any matrix, was used with each batch of samples analysed.

#### 2.2.3. Virus Detection by RT-qPCR

HEV and the Rotavirus SA11 (SPCV) were tested using one-step duplex reverse transcription real-time PCR (RT-qPCR) following the oligonucleotides, controls and conditions previously described [13,14,15,16,17,18,19]. All RT-qPCRs included an internal amplification control (IAC), constructed as described by Diez-Valcarce et al. [14]. All RT-qPCRs were conducted in a duplex format, targeting the specific viruses (HEV or SPCV) with a FAM-labeled probe and the chimerical internal amplification control (IAC), using a VIC-labeled probe. The thermocycling conditions were as described previously [20]: 15 min at 50 °C, 2 min at 95 °C, followed by 40 cycles of 15 s at 95 °C and 1 min at 60 °C. The extracted RNAs were tested undiluted and ten-fold diluted to reduce the inhibitory effect in the samples.

#### 2.2.4. Reporting and Interpretation of RT-qPCR Data

Four different signals were considered for the correct interpretation of the results: (i) the target virus; (ii) the SPCV virus; (iii) the target IAC; (iv) the SPCV IAC [11,12,14]. When a RT-qPCR assay showed a HEV Cq (quantification cycle) value ≤ 40, independently of the corresponding IAC Cq value, the result was interpreted as positive. When an assay showed a HEV Cq value ≥ 40, i.e., no amplification was observed, but signals for HEV IAC, SPCV and its IAC were present, the absence of target virus signal was conclusively considered as a test negative result.

#### 2.2.5. Extraction Efficiency

The extraction efficiency was calculated by comparing the SPCV Cq value of the sample with the SPCV Cq value of the NPC, using the following formula: 2 ^(Cq TNPC − Cq sample)^ × 100 [17]. Efficiency results were classified as insufficient (extraction efficiency <1%), acceptable (1–5%), good (5–25%) and very good (>25%).

### 2.3. Statistical Analyses

A nonparametric analysis of variance (Kruskal-Wallis) performed with Statistic 7.0 was used to evaluate the presence of differences in viruses parameters obtained from the samples collected on different sites and seasons. A Pearson correlation and linear regression test, ANOVA test, and Student’s *t*-test were performed using GraphPad Prism 5.0 (GraphPad Software Inc., San Diego, CA, USA); data were considered statistically significant at *p*-value ≤ 0.05.

## 3. Results

### 3.1. Efficiencies of Virus Concentration and RNA Extraction Form Manure Samples

The mean virus extraction efficiency of the process was 4.2% with a standard error of 0.5%. Values ranged from 1.9% to 7.9%. Overall, 58% of the manure samples showed acceptable extraction efficiency (1–5%), and 42% showed good extraction efficiencies (5–25%).

### 3.2. HEV Detection in Swine Farms

**Figure 1:**
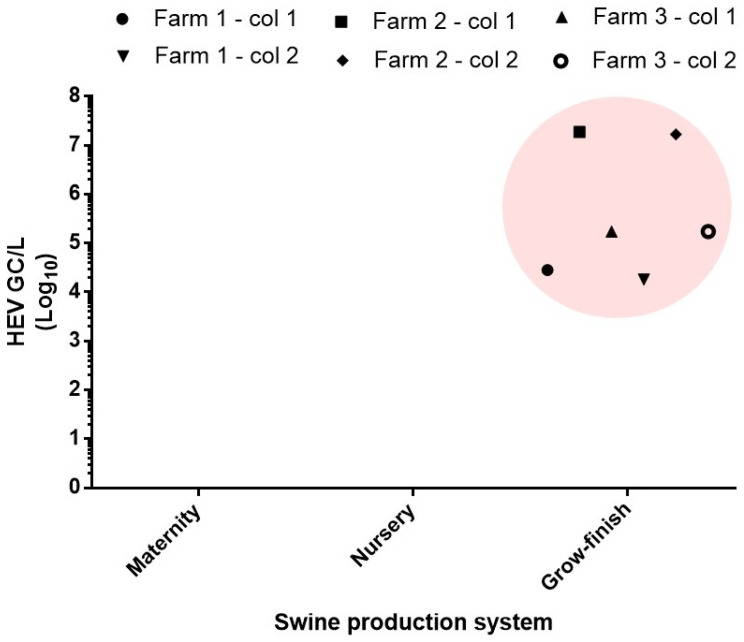
HEV concentration in swine raw manure from maternity, nursery and grow-finish swine production system. GC: genome copies.

Swine manure from different types of farms (swine maternity, swine nursery, and grow-finish swine production) was evaluated for the presence and quantification of HEV. HEV was consistently detected in raw manure from the three grow-finish farms, and the viral quantities were consistent along the two years evaluated, pointing to persistent contamination (Figure 1; Table 1). However, while HEV was detected in raw manure from grow-finish swine production, it was not detected in maternity and nursery farms (Figure 1).

### 3.3. HEV Reduction in Swine Manure after PABs

All the raw manure samples of the three pig growing-finishing farms were positive for HEV. We further evaluated the efficiency of PABs to remove HEV from swine manure, by determining the HEV concentration in effluent water after PAB treatment. The reduction in HEV load is summarized in Table 1. Reduction ranged from 1.40 to 3.00 log_10_ HEV genome copies L^−1^ (94.63% to 99.90% reduction), with an average reduction of 2.23 log_10_ HEV genome copies L^−1^ (99.41% reduction). However, the HEV load in the final effluent was still high, ranging from 2.05 to 5.24 log_10_ HEV genome copies L^−1^, with an average of 3.85 log_10_ HEV genome copies L^−1^.

## 4. Discussion

We evaluated the presence of HEV in manure at different stage of pig production. HEV was consistently detected in the pig grow-finish farms and the viral quantities were constant along the two years monitored, pointing to persistent contamination. However, manure from maternity and nursery swine farms was negative for HEV. This is an interesting finding, as first contact with HEV usually occurs after weaning (22 to 29 weeks of age) [21] and therefore our results are consistent with high biosafety control during primary-swine production. This finding is in agreement with those shown previously in Brazil: De Souza et al. [22] found HEV in feces, serum, and liver from swine at slaughter age (approximately six months) in the state of Pará, Brazil, but the HEV positive samples were from animals without serological evidence of HEV infection (IgM or IgG), also suggesting that the biosafety procedures applied in swine nursery in that study were effective for HEV infection, similarly to that observed in the present study.

Environmental contamination is a relevant transmission route for HEV. Parashar et al. [23] detected HEV by RT-qPCR in soil until at least nine weeks. Andraud et al. [24] demonstrated that the environmental HEV load is important route for domestic pig contamination: infected animals may excrete more than 106 HEV GC g^−1^ of feces, but inocula with titres higher than 10^6^ HEV GC L^−1^ are required for efficient infection of animals and promotion of virus spread. In the present study, high HEV loads (>6 log_10_ HEV GC L^−1^) in swine raw manure of pig grow-finish farms were observed, representing a significant risk of contamination for the animals of the herd. Although HEV is more frequently detected in feces than serum and liver samples from infected animals [16,20,22], the foodborne hepatitis E risk through consumption of pig meat or derived products cannot be discounted.

Considering the importance of controlling HEV environmental contamination by sanitation in swine farms, we investigated the ability of PABs to reduce the HEV load after anaerobic biodigestion. Previous studies by our research group had obtained divergent anaerobic biodigestion inactivation results regarding the virus types examined. While PABs operating at 24 °C showed an unsatisfactory reduction of porcine circovirus and porcine adenovirus, rotavirus A load was reduced by more than 90% [25]. The results obtained in this study showed a reduction higher than 99% (>2 log_10_ HEV GC L^−1^) in treated effluents compared to raw manure, suggesting an efficient HEV load reduction. PABs can be an effective system for reduction of pathogen loads, but the need remains for sanitary surveillance and legislation of swine manure-derived biofertilizers to avoid the spread of zoonotic enteric pathogens, such as HEV, and recontamination into the farms. The use of PABs combined with strict biosafety and hygiene protocols can contribute to better microbiological standards in swine products.

## 5. Conclusions

Our study demonstrated the presence of HEV in manure in pig farms. While manure form maternity and nursery pig farms was negative for HEV, manure from grow-finish farms was consistently positive for HEV. This shows a potential high biosafety status during primary-swine production, with subsequent virus contamination in grow-finish production. Our study also demonstrated that PAB can be a robust tool for effective inactivation of HEV (>99.00% reduction), showing better virus reduction than for other porcine pathogenic viruses such as porcine circovirus.

## Figures and Tables

**Table 1:** HEV concentration in swine raw manure and swine effluent after digestion in psychrophilic anaerobic biodigesters, as well the reduction after treatment. GC: genome copies. Values are represented as Mean ± Standard Error.

	Year	Farm 1	Farm 2	Farm 3	Average
HEV in Raw Manure (log GC L^−1^)	2018	4.45 ± 0.9	7.27 ± 0.02	6.72 ± 0.10	
	2019	4.26 ± 0.12	7.22 ± 0.03	6.51± 0.13	
	Average	4.36 ± 0.10	7.25 ± 0.02	6.63 ± 0.11	
HEV in Final Effluent (log GC L^−1^)	2018	2.08 ± 0.01	4.27 ± 0.01	5.23 ± 0.02	
	2019	2.05 ± 0.02	4.22 ± 0.03	5.24 ± 0.01	
	Average	2.06 ± 0.01	4.25 ± 0.02	5.23 ± 0.01	
Reduction (log GC L^−1^) (%)	2018	2.37 ± 0.11 (99.57%)	3.00 ± 0.01 (99.90%)	1.46 ± 0.13 (96.79%)	2.29 ± 0.53 (98.76%)
	2019	2.21 ± 0.09 (99.38%)	3.00 ± 0.01 (99.90%)	1.27 ± 0.08 (94.63%)	2.16 ± 0.61 (97.97%)
	Average	2.30 ± 0.08 (99.50%)	3.00 ± 0.01 (99.90%)	1.40 ± 0.11 (95.98%)	2.23 ± 0.52 (99.41%)
